# Correction to: Clinical testing on SARS-CoV-2 swab samples using reverse-transcription loop-mediated isothermal amplification (RT-LAMP) & Two extraction-free reverse transcription loop-mediated isothermal amplification assays for detection of SARS-CoV-2

**DOI:** 10.1186/s12879-023-08738-3

**Published:** 2023-11-16

**Authors:** Yee Ling Lau

**Affiliations:** Department of Parasitology, Faculty of Medicine, University of Malaya, Kuala Lumpur, 50603 Malaysia

**Correction to**: ***BMC Infect Dis*22, 697 (2022)**

10.1186/s12879-022-07684-w

**Correction to**: ***BMC Infect Dis*21, 1162 (2021)**

10.1186/s12879-021-06876-0

The original publications of these articles contained an incorrect funding note. The incorrect and correct information is listed in this correction article, the original article has been updated.

**Incorrect**

- This study was supported by Prototype Research Grant Scheme (PRGS), PR001-2020B from the Ministry of Education, Malaysia.
- This study was supported by Prototype Research Grant Scheme (PRGS), PR001-2020B (PRGS/2/2020/SKK09/UM/02/1) from the Ministry of Education, Malaysia.

**Correct**

- This study was supported by Prototype Research Grant Scheme (PRGS), PR001-2020B (PRGS/2/2020/SKK09/UM/02/1) from the Ministry of Higher Education, Malaysia.
- This study was supported by Prototype Research Grant Scheme (PRGS), PR001-2020B (PRGS/2/2020/SKK09/UM/02/1) from the Ministry of Higher Education, Malaysia.

